# The Effects of Cryotherapy on Proprioception System

**DOI:** 10.1155/2014/696397

**Published:** 2014-11-12

**Authors:** Mariusz Paweł Furmanek, Kajetan Słomka, Grzegorz Juras

**Affiliations:** The Jerzy Kukuczka Academy of Physical Education in Katowice, Department of Human Motor Behavior, 72A Mikolowska Street, 40-065 Katowice, Poland

## Abstract

Proprioception plays an important role in the complex mechanism of joint control. Contemporary sport activities impose extremely high physical demands on athletes. Winter sports are played in areas with excessively low temperatures. Moreover, many athletes are subjected to treatments that involve local lowering of the body temperature before, during, and after physical activity. This work reviews the current knowledge regarding the influence of local cryotherapy on the proprioception system. The reviewed literature identified several tests that evaluate different aspects of proprioception. There is no universally agreed protocol, or clear set of criteria for test conditions. The outcomes of different tests and assessments of cryotherapy procedures using different cold modalities are poorly correlated. In general, the published results on the mechanism of cryotherapy effects on proprioception are not uniquely conclusive and are frequently contradictory. Additional high-quality research is required to explicitly answer the following questions: (1) whether local cryotherapy influences all aspects of proprioception; (2) whether the current methods of evaluation are adequate for the exploration of the relationship between cryotherapy and proprioception; and (3) whether the application of local cryotherapy is safe for athletes regarding proprioception. The review clearly showed that there is no comprehensive model relating cryotherapy and proprioception.

## 1. Introduction

The term “proprioception” was developed in 1906 by Sherrington [1], who first used the hypotheses of the “proprioceptive field,” the “proprioceptive reflex,” and the “proprioceptive system.” Proprioception is defined as the perception of joint position and movement as well as “the* afferent information arising from internal peripheral areas of the body* (located predominantly in the muscles, tendons, joint capsules and ligaments)* that contribute to postural control* (postural equilibrium),* joint stability* (segmental posture),* and several conscious sensations* (muscle sense)” [2, 3]. Proprioception is very important in neuromuscular control and is considered to be a subset of the entire somatosensory system [4–6]. Joint position sense, kinesthesia, and a sense of force (tension, resistance, or weight) comprise proprioception [3, 7–9]. Kinesthesia as a subcomponent of proprioception should not be used as a synonym for proprioception. Postural equilibrium, which describes the balanced state of forces and their moments acting on the center of the body mass, is primarily based on vestibular information (to be proprioceptive with respect to the head); additionally, it depends on muscle sense, joint position sense, and resistance to movement. Proprioception and balance are interconnected [10], and, for the purpose of this review, balance is considered with proprioception.

Neuromuscular control is based on subconscious information from mechanoreceptors and processes within the central nervous system that allow control movement through coordinated muscle activity. It results from a complex interaction between the nervous system and the musculoskeletal system through feedback and feedforward mechanism control [5]. Riemann and Lephart [6] suggested that the role of proprioceptive information in motor control could be separated into two categories. The first category is the role with respect to the external environment (unexpected perturbation), and the second category concerns the planning and modification of internally generated motor commands. Proprioception inputs are critical, particularly for athletes and in daily activities and physiotherapy. First, proprioception prevents an excessive range of motion through the proprioceptive reflex. Second, deep feeling is required to stabilize joints within the body during static (e.g., posture) positions and dynamic movement (e.g., gait). Third, proprioception plays an important role in the complex process of coordination and in the precise movement of joints to prevent joint damage. Finally, proprioception constitutes the basis of each neuromuscular and coordination training plan [5, 11, 12].

To avoid the misuse of the components of proprioception and evaluate the correct variables during investigations into proprioception, these definitions are reviewed in this paper. Joint position sense determines the ability of a subject to perceive a required (desired) joint angle [13–18]. Kinesthesia (the sense of movement) refers to the ability to distinguish joint movements, including the duration, direction, amplitude, speed, acceleration, and timing of movement [18–23]. The sense of force represents the ability to differentiate force generated within the joint [24, 25]. Balance is the process of maintaining the center of gravity within the base support of the body [26].

The following three major testing procedures for the proprioception system are described in the literature, depending on which submodality is tested: (1) the reproduction of positioning, commonly called joint position sense (JPS), (2) the threshold of the detection of passive motion (TTDPM), and (3) the force (re)production sense (FR). In the JPS test, the joint is moved (actively or passively) towards the earlier requested (reference) angle. After a few seconds, the joint is returned to the original position. Following this movement, the subject should reproduce the perceived angle with the identical or contralateral knee or, in some cases, demonstrate the perceived angle on the joint model [12, 21, 27, 28]. In the TTDPM test, the joint is slowly and passively moved (0.5–2°/s). The subject is required to detect stop/start and several parameters (see above) of this movement as quickly as possible. Frequently, the subjects are required to name the joint that is moved [12, 19, 22, 23]. In the FR test, the subjects are required to discriminate weight that is actively lifted, pressed, pinched, or gripped. Additionally, force reproduction involves the use of a reference force, typically determined as a percentage of a maximal voluntary isometric contraction (MIVC) and an attempt to replicate that force. Force matching occurs in the identical limb or the contralateral limb [7, 24, 25, 29–31].

Contemporary sports activities impose extremely high physical demands on athletes. Many of them are subjected to treatments that involve local changes in body temperature to obtain therapeutic effects. Cryotherapy consists of the lowering of tissue temperature by withdrawing heat from the body to achieve an analgesic effect [32]. Superficial and deep temperature changes depend on the application procedure, initial temperature, application time, application area, and location [33]. The most common cold media are ice (packaged crushed ice), cold water (water immersion), cooling pads (gel packs), cold air, evaporating spray, or vaporized liquid nitrogen [34–36]. Additionally, the final method is used in a form of cryostimulation in cases in which the temperature of the cooling medium is lowered to below −100°C [37, 38]. Cryotherapy, depending on the procedure, reduces pain, edema, inflammation, tissue temperature, metabolism, muscle stiffness, and nerve conduction velocity. Cold is found to decrease cellular metabolism, which helps reduce the extent of secondary injury. Cold is not only applied for the treatment of acute and chronic soft tissue injuries. Some studies have shown benefits to athletes after both local and whole body cryotherapy [39–43]. The application of cold increases the pain threshold, tissue viscosity, production of endorphins, testosterone, readiness for physical activity, and general recovery from fatigue and stressful bouts of sports training [44]. In that context, many sports competitions are in locations with excessively low temperatures.

Though it is relatively simple to use cryotherapy by decreasing the tissue temperature, much controversy and confusion remain regarding the clinical practice, particularly in the literature on the influence of cryotherapy on the proprioception system. This controversy was reported in the most recent review of Costello and Donnelly [45]. This specific review is limited to the JPS aspect of proprioception. In addition, conclusive scientific research is lacking regarding the potential risks or benefits of using cryotherapy [46, 47]. Despite this lack of knowledge, coaches, physiotherapists, and athletes frequently use cryotherapy before, during, and after physical performance.

The purpose of this review is to answer questions regarding (1) the effects of locally applied cryotherapy on each aspect of proprioception system, (2) the appropriateness of today's methods of proprioception assessment, and (3) the safety of local cryotherapy for athletes when proprioception is considerate. The first two questions are of fundamental importance for the proprioception studies, while the third is more specific and narrow.

The primary objective of this paper was to review the literature in the context of the relationship between cryotherapy and the proprioception system. The effects of local cooling on functional performance and other factors influencing proprioception, including aging, fatigue, warm-up, regular physical activity, and exercise, are found in comprehensive review papers by Ribeiro and Oliveira [9] and Bleakley et al. [47].

## 2. Methods

### 2.1. Search Methods

The field of human kinetics was selected for a search of original research contributions. In addition, the fields of medicine and sports medicine were examined. A search was conducted using the following databases: PubMed, SPORTDiscus, Scopus, EBSCOHost, ScienceDirect, and Web of Science. Keywords including cryotherapy, cooling, cold packs, ice, gel packs, proprioception, joint position sense, kinesthesia, sense of force, proprioception system, and balance were used in various configurations. The search period regarding the relationship between proprioception and cryotherapy was observed from January 1990 to December 2013.

### 2.2. Selection Criteria

The criteria for the study selection were as follows: the publication language was English; the subjects were young adults, humans, and healthy; tests were conducted within at least five minutes after the application of cryotherapy; and cryotherapy was applied locally. The outcome measures were all aspects of proprioception, including balance, joint position sense, kinesthesia, and force sense. Studies regarding the effects of local cooling on maximal functional performance were excluded from this review.

### 2.3. Synthesis of Methods and Procedures

The summary and details of the methods and procedures used in the reviewed publication are listed in Table 1. Seventeen studies that involved 398 healthy subjects qualified for the review. The mean number of participants per study was 23.4 ± 10 with a mean age of 22.8 ± 2.5 years. At least 155 female and 171 male subjects took part in the data collection (the sex of 72 participants was not declared, Ingersoll et al. [48], LaRiviere and Osternig [49], and Douglas et al. [50]). The most frequently examined parts of the body and place of cryotherapy application were as follows: knee (7), ankle (6), foot (3), shoulder (2), thigh/quadriceps muscle (2), lower limbs (1), and hand (1). The following components of proprioception were assessed: active and passive joint position sense, 11 studies [15, 17, 49, 51–58]; balance, 5 studies [48, 50, 55, 59, 60]; and force sense, 2 studies [24, 29]. None of the published articles investigated kinesthesia as the threshold of detection of passive motion. Typically, researchers assessed the dominant extremity (9 studies) because differences are rarely found between the right and the left legs during the testing of proprioception, for example, [23, 61]. One team assessed a limb selected by a subject [54]. In one investigation, the extremities were selected randomly [52]. Only left extremities were assessed in one study [51], whereas Ingersoll et al. only assessed right extremities [48]. Three teams investigated both limbs during testing [24, 53, 59].

The average time of cryotherapy application was up to 20.3 ± 5.3 minutes and depended on the cooling medium. Ice (mean time 21.4 ± 3.8 min) or cold water immersion (mean time 18.6 ± 5.6 min, mean temp. 6.6 ± 4.7°C) was used. One team cooled the tissue as long as required for the muscle temperature to decrease to 3°C. Seven teams used water immersion, and seven used ice compresses. One group used commercial cold packs, one group used an icing system, and one group used an ice blanket consisting of sacks filled with crushed ice (Table 1, cryotherapy procedure). All 17 studies set the level of statistical significance at *P* < 0.05 and compared the posttreatment results with the baseline or a control group.

## 3. The Effects of Cryotherapy on Joint Position Sense in Healthy Participants

Joint position sense (JPS) was the most frequently investigated aspect of the proprioception system, with eleven of the eligible studies focusing on JPS. The review of this literature revealed that cryotherapy had a negative effect on the JPS in four studies [52, 53, 55, 56]. Seven studies showed that cooling has no influence on the JPS [15, 17, 49, 51, 54, 57, 58]. Because each team used a different JPS test and a different cryotherapy procedure, comparing and reconciling those results are difficult. The major conclusion from three of the four studies showed a deleterious effect on JPS. Oliveira et al. [56] found that there is no significance if one cools the quadriceps muscle or only the knee joint (*P* = 0.958). Uchio et al. [53] demonstrated that the knee joint becomes stiffer after cryotherapy (laxity decreased, *P* = 0.003). Additionally, Surenkok et al. [55] found that the pain threshold after cryotherapy significantly changed (*P* < 0.005). These authors unanimously suggested that caution should be exercised if cryotherapy is used immediately after physical activity. Hopper et al. [52], who obtained a reduction in JPS of 0.5° ± 0.75° (*P* = 0.049) after cold-water immersion (4°C) of the ankle joint, concluded that these results should not be deemed clinically significant.

None of the remaining seven papers reported any significant changes in the JPS (expressed as a variety of angular errors in degrees or positional errors in centimeters). The results of those studies showed that there is no evidence of an increased risk of injury following a return to exercise after cryotherapy. Though no differences were found for active JPS after cryotherapy application, Wassinger et al. [17] concluded that neuromuscular deficits could potentially occur and impair the athlete's functional ability in the short term. This conclusion was derived because, in addition to JPS, they measured a path of motion replication and conducted a functional throwing performance test.

Based on the above conflicting evidence, it is very difficult to categorically state whether cryotherapy impairs the joint position sense. Some authors [17, 55, 58] demonstrated several procedural limitations, and the results should be interpreted with caution.

## 4. The Effects of Cryotherapy on Kinesthesia in Healthy Participants

In the literature, there are a number of papers regarding kinesthesia [18–23]. None of these studies reported on the mechanism of the influence of cryotherapy on kinesthesia. One study [51] investigated the effect of cryotherapy on the movement reproduction pattern (timing and accuracy) and the angle reproduction (peak angle, final angle). The authors did not observe the threshold of the detection of movement, which is a commonly accepted method of kinesthesia evaluation. Regardless of the evaluation method used, the authors did not observe any adverse effect on proprioception.

## 5. The Effects of Cryotherapy on Force Sense in Healthy Participants

In a review of the literature, two studies investigated the influence of cryotherapy on the force sense as one of proprioception aspects [24, 29]. Rubley et al. [24] measured the force sensation in the distal palmar aspect of the index finger and thumb. They supplemented the investigation by adding the sensation of pressure and two-point discrimination tests. The force sense required the subjects to match as accurately as possible (5 trials of 30-s duration each) isometric target forces of 10, 25, and 40% of MVIC (maximal voluntary isometric contraction). The root mean square error during each trial, which represented the subject's accuracy, was calculated. In a team from Canada, Tremblay et al. [29] checked the thigh muscle sense (of the perception of force and weight) using a task that they called weight-discrimination. The test consisted of one set of 14 trials. They used a standard weight and a reference weight. The participants lifted each weight successively and reported which weight was heavier sequentially. The results (the number of correct discriminations) were calculated as a performance value in a percentage.

The studies concluded that the perception of force signal is not affected by local cooling, though the studies used different cryotherapy procedures (Table 1) and targeted different parts of the body. The authors of these studies unanimously support the hypothesis that the application of cold is not contraindicated for use before exercises focusing on restoring neuromuscular control.

## 6. The Effects of Cryotherapy on Balance Sense in Healthy Participants

Relatively few papers have been published regarding the influence of local cryotherapy on balance sense [48, 50, 55, 59, 60]. Douglas et al. [50] investigated static and dynamic conditions and found a statistically significant increase only in the mediolateral component of a dynamic balance test following ice water immersion (temperature below 4.4°C). Williams et al. [60] conducted the following two tests: a star excursion balance test as a dynamic or functional test and a static balance test using a force plate. They did not observe any difference after the application of local cooling. Dewhurst et al. [59] assessed two quiet standing positions with a large and narrow support base with open and closed eyes. Cooling of both legs until the temperature of the muscles decreased by 3°C did not affect the postural assessment. Surenkok et al. [55] conducted a balance test to complement the JPS investigation following a cold commercial gel-pack and a cold spray application. The interesting outcome of the study was a significant difference in the one-leg static balance test and only for cold pack application. Ingersoll et al. [48] assessed postural balance using a one-leg balance test (stork stand); the length of time for which the subject maintained this posture was used as the measure of balance. Additionally, this team measured topagnosis and administered two-point discrimination tests. None of the dependent variables were changed significantly after cryotherapy. 

## 7. General Conclusions from the Reviewed Studies

Twelve studies reported no adverse effects on proprioception [15, 17, 24, 29, 48, 49, 51, 54, 57–60]. In the remaining five investigations, cryotherapy had a negative (harmful) effect on proprioception [50, 52, 53, 55, 56].

## 8. Discussion

Our discussion of the reviewed papers focused on addressing the three questions stated in the introduction. Specifically, we tried to determine the following: (1) whether locally applied cryotherapy affects all aspects of the proprioception system; (2) whether the current methods of proprioception evaluation are adequate for exploring the relationship between cryotherapy and the proprioception system; and (3) the safety of the use of cryotherapy by athletes in cases in which proprioception is considered.

The critical review of the literature clearly shows contradicting evidence on the influence of local cryotherapy on proprioception. A number of researchers [17, 34, 45, 53, 55, 56] maintain that local cryotherapy impairs deep feeling in the body, and they unanimously conclude that neuromuscular deficits occur after cold application and advise athletes against undertaking dynamic training immediately after cryotherapy. This view was reinforced by an investigation by Herrera et al. [62], who assessed the nerve conduction velocity (NCV) after ice pack application (crushed ice) for 15 min. They recorded decreased sensory and motor NCV by 16.7 m/s and 2.1 m/s, respectively. A similar investigation was conducted by Algafly and George [63], whose data showed that, after the application of an ice bag filled with crushed ice, NCV was significantly reduced by 32.8% (*P* < 0.05). With theprogression of skin temperature lowering to 10°C, the pain threshold and pain tolerance increased (*P* < 0.05). In contrast, several investigators [15, 24, 29, 48–51, 54, 57–60] found no changes in the proprioception system and no evidence of an increased risk of injury following a return to athletic activity after cryotherapy. Cold water immersion of the ankle joint at a temperature of 4–6 ± 1°C for 5, 15, and 20 minutes did not significantly influence the JPS of the ankle joint [49, 58]. Cold water immersion (1°C) of the foot and ankle joint did not affect sensory perception [48]. Water immersion (temperature 14 ± 1°C) of 30 min duration to the level of the subject's umbilicus did not significantly change the non-weight-bearing or weight-bearing assessment of knee joint position sense [57]. Similar results emerged from an investigation of the shoulder joint after the application of a cubed ice compress for 30 min [15]. The investigators who applied a variety of cold packs (cubed, crushed ice) in the knee joint region found no influence on proprioception in this particular joint [51, 54]. Hart et al. [54] concluded, based on EMG data, that knee joint cryotherapy (20 min, cubed ice) does not place the lower extremity at a risk of injury during landing strategy (the control of joint stability was not adversely changed after cooling).

Other aspects of the proprioception system such as balance and force sense have been investigated. These characteristics of proprioception appear to be the least influenced by cryotherapy. Douglas et al. [50] obtained results suggesting that cryotherapy of the ankle has a negative effect on the mediolateral component of dynamic balance; however, those results should be interpreted with caution. Other investigators [24, 29, 59, 60] reported that cryotherapy is safe for neuromuscular control in healthy young adults. Even in a group of elderly women 73 ± 3 years of age, cryotherapy did not affect postural steadiness [59].

This review found that only five studies [17, 50, 53, 55, 56] concluded that, regardless of the cryotherapy procedure used, at least one test delivered a negative effect on proprioception. The authors of these studies report the limitations of their studies; for example, only one of the five research teams measured the surface skin temperature of the investigated joint [53], and knowing this temperature is crucial to determining the effectiveness of cooling. In a study by Douglas et al. [50], the 10-s trials of a dynamic balance test are questionable, and the subjects were tested exclusively with the open-eyes condition. According to Barrack et al. [64], Lephart et al. [65], and Olsson et al. [66], proprioception testing should exclude the visual, hearing, and touching sensory receptors to rely more on mechanoreceptors such as muscle spindles, Golgi tendon organs, and vestibular receptors. Wassinger et al. [17] noted that their test condition might not represent an exact alignment with the normal motion of subjects, which, in turn, could affect their results. These findings should be treated with caution.

Twelve of the seventeen studies indicate that there is no evidence for a deleterious effect by cryotherapy on the proprioception system including JPS, force sense, and balance.

McCloskey [67] postulated that the sense of passive movement and direction of movement might constitute a mechanism of proprioception and must be considered with movement being detected before recognizing the direction of movement. Measurements of the threshold to the detection of passive movement (TTDPM) are the most reliable and validated method of measuring the kinesthetic aspect of proprioception [21, 68]. This review clearly showed that TTDPM measurements present a challenge typically because of a lack of a specifically designed research apparatus with slow angular velocity 0.5–2°/s [22]. Cryotherapy adds further difficulties to the measurement procedure, for example, a limited data acquisition time after the cold application. This review demonstrates the following: (a) the large deficit in our understanding of the relationship between kinesthesia and cryotherapy and (b) the lack of adequate tools and instruments to measure kinesthesia under cryotherapy conditions. This field presents a significant potential for future scientific work.

Many studies report that laboratory investigations on proprioception seldom reflect the actual load demands during functional movement (during sports or daily activities). Costello and Donnelly [57] proposed a protocol to overcome these deficits. They measured active ipsilateral limb repositioning sense using an accurate technique for weight-bearing and non-weight-bearing JPS assessment. They did not observe any significant differences after cryotherapy. In their earlier comprehensive review regarding the influence of cryotherapy on JPS, they cautioned against returning to dynamic activities immediately after cooling.

Additionally, during the local application of cryotherapy, including treatment of one part of the joint or muscle (e.g., the anterior aspect of the knee joint and extensor muscle), proprioception could be partially compensated for by other receptors such as ligament receptors or flexor muscle receptors. During an investigation using ice water immersion as a cold modality (e.g., Costello and Donnelly [57]), all of the responsible receptors were affected in a similar manner.

Of the seven reviewed studies in which the investigators used cold/ice water immersion, only two obtained a significant influence on proprioception [50, 52], and both results are questionable. The questions remain regarding the duration of the effects of joint and muscle cooling and the depth that cryotherapy reaches. There are studies regarding the intra-articular and inner muscle temperature [59, 69, 70]. The muscle temperature was measured using a flexible temperature probe inserted 1 cm below the subcutaneous fat layer at an angle of 45° in the direction of the muscle fibers [59]. The intra-articular temperature was measured in the cavity of the knee joint using a spinal needle [69] or an 18-gauge needle [70]. The muscle and intra-articular temperatures were significantly lowered. Even after two hours, the intra-articular temperatures did not return to their baseline level [69, 70]. This research presented evidence that cooling has a deep and lasting effect. Daily physiotherapeutic practice should be considered. Frequently, cryotherapy is applied only to a limited part of the joint or muscle and over a limited period of time. The selection of a cryotherapy application procedure is important because different cooling treatment regimes might yield different results. Clinical practice should have a standard for establishing correct treatment modalities and durations.

The investigation of proprioception allows an estimation in the subjects of the actual level of the neuromuscular system functionality, the participation of the neuromuscular system in joint stabilization, the magnitude of damage to the neuromuscular system functioning, and the physiotherapy progression and its efficiency. The following key factors should be considered in proprioception investigations and the analysis and interpretation of the data to obtain reliable and validated databases: the precision of the measuring device (instrument) and measurement error; the aspects of proprioception; the method of measuring (active, passive); the quantity of the measurements; the methods of averaging; the investigated variables (the absolute, relative, and variable errors); the units of variable (degree, millimeter); the elimination of external factors that influence proprioception (sight, hearing, cutaneous sensation, and other feedback); the investigated joint; the starting position; the gravitational effects (weight-bearing, non-weight-bearing); the movement direction; and the movement velocity. Considering the above factors, the results of the reviewed papers should be interpreted with care.

## 9. Conclusions

The reviewed literature identified several tests that evaluate different aspects of proprioception. There is no standardized protocol or clear set of criteria for those test conditions. The outcomes of the tests and the cryotherapy procedures using different cold modalities are poorly correlated.

This review clearly showed that there is no comprehensive model relating cryotherapy and proprioception, and the results from the proprioception assessment methods provide a limited view of the manner in which cryotherapy and proprioception are related.

The most frequently investigated aspect of proprioception was JPS, which is perceived to be one of the more functional tests for proprioception evaluation. In the published literature, there are no publications concerning the relationship between kinesthesia and cryotherapy. Two other aspects of proprioception, balance, and force sense appeared to be unaffected by cryotherapy.

The reviewed literature does not provide conclusive evidence regarding the safety of local cryotherapy use for athletes or the evaluation of proprioception.

The following general conclusion was determined in the study: a novel, standardized protocol, better evidence, and better quality studies regarding the relationship between proprioception and local cryotherapy should be conducted on a broader range of samples to answer unequivocally the major areas of investigation of this study.

## 10. Recommendations for Future Research

Future research should concentrate on establishing a functional relationship between the proprioceptive system and local/entire body cryotherapy. To achieve this objective, the following actions are needed:

- propose a priority list of proprioception aspects that should be evaluated when the individual subject condition is to be considered (differentiation between athletes and patients);
- establish a standardized protocol for proprioception evaluation. The outcomes of tests for the aspects of proprioception poorly correlate with each other. The proposed procedure should attract greater attention to proprioception evaluation in other parts of the body, including the elbow, wrist, hip, and spine. The proposed procedure should include rotational and sectional body movement when testing proprioception;
- design and build a new and comprehensive instrument for measuring the aspects of proprioception (JPS, TTDPM, FR), which will be sensitive and reliable and will limit the risk of bias;
- specify the required duration and spatial extent of local cryotherapy to achieve a specific penetration depth of treatment in cases in which intramuscular and joint cooling studies are conducted;
- extend the proprioception investigation to a broader range of initial conditions regarding the conditions of the subjects, for example, large groups of subjects, clinical groups, elite athletes, and children;
- conduct proprioception investigations at various times in the rehabilitation process;
- establish a multifaceted training regime of the proprioception system;
- assess the possibility of introducing a placebo effect into the investigation of proprioception.

## Figures and Tables

**Table 1:** Summary of publications that investigated the effect of cryotherapy on proprioception system (according to date of publication).

Authors	Cryotherapy procedure	Area	Subjects	Proprioceptive test	Instrument	*P* value
Douglas et al., 2013 [50]	15 min ice water immersion Temp.: below 4.4°C at the level of 5 cm above the medial malleolus Data collection: before and immediately after	Foot and ankle dominant extremity	*n* = 20 Age: 23.9 ± 2	Balance test static and dynamic 3 × 10 s V-stability A/P V-stability M/L V-overall stability (OA)	Biodex Balance System^a^	Static balance *P* > 0.05 for all variables Dynamic balance AP and OA *P* > 0.05 ML **P** = 0.026

Williams et al., 2013 [60]	20 min crushed ice, 1.1 kg 2 different conditions ice bag with compression, ice bag without compression 72-hour rest interval	Ankle dominant leg	*n* = 30 M—9 F—21 Age: 20.6 ± 1	Star excursion balance test reaching nondominant leg as far as possible V-rich distance, normalized [%] Static balance, single leg 10 s period V-path and velocity of COP	Force plate^b^	*P* > 0.05 for all variables

Costello and Donnelly, 2011 [57]	30 min water immersion Temp.: cold water (14 ± 1°C) tepid water (28 ± 1°C) participants were seated in a water immersed to the level of umbilicus Data collection: before and after	Knee dominant extremity	*n* = 14 M—8 F—6 Age: 23.3 ± 1	A-JPS non-weight-bearing P: seated position with the leg 90° flexion of knee ~35°, 55°, and 75° randomly assigned index angle ~5 s hold and reproduced weight-bearing P: stand with 95% approx. of body weight on testing leg Flexion (~60°) to Extension (~45°) extension (0°) to flexion (45°) 3 trials for each angle Blindfolded V-absolute error, relative error, variable error [°]	Eagle 3D Motion System^c^ 3 markers: (i) greater trochanter, (ii) lateral epicondyle of the femur, (iii) lateral malleolus *f* = 400 Hz	*P* > 0.05 for all variables

Khanmohammadi et al., 2011 [58]	15 min water immersion Temp.: 6 ± 1°C 5 cm above the malleolus Data collection: before, after, and 15 min after	Ankle dominant extremity	*n* = 30 F—30 Age: 21.9 ± 0.8	A-JPS and P-JPS P: seated from neutral position to the middle range of dorsal-flexion at 10° and 20° of plantar-flexion, first passive and then active time to memorize 3 s Blindfolded, V-error [°]	Goniometer	*P* > 0.05 for all variables

Oliveira et al., 2010 [56]	20 min crushed ice 1200 g Ice bag: 20 × 25 cm Data collection: before and immediately after	Knee, quadriceps muscle dominant extremity	*n* = 15 M—6 F—9 Age: 22.4 ± 1.4	A-JPS reproduction of 7 angles between 40° and 60°, 3 trials *OKC* P: seated 90° flexion of knee, movement to extension with velocity ~10°/s, time to memorize 5 s Blindfolded V-absolute error, relative error [°]	4 markers: (i) greater trochanter, (ii) lateral epicondyle of the femur, (iii) lateral malleolus, (iv) neck of fibula Video images analysis^d^	**P** = 0.015 for all variables no difference due to cryotherapy location

Surenkok et al., 2008 [55]	30 min cold pack (3M cold/hot gel pack) center of the gel pack over the tip of the patella 1 week later, following cold spray application Data collection: before, after balance test, 5 min after	Knee dominant extremity	*n* = 15 M—15 Age: 22 ± 2.8	P-JPS P: seated 90° flexion of knee movement velocity 5°/s, towards flexion or extension Test: press stop button in the middle of the range of motion-45° Blindfolded, 3 trials V-absolute error [°] Balance test V-balance index-BI	Cybex dynamometer^e^ Movable platform^f^	**P** < 0.05 for all variables but not for cold spray in case of balance test

Dewhurst et al., 2007 [59]	As long as temp. decrease 3°C with respect to control muscle temp. Specifically made ice blanket consisting of large, thick plastic sacks filled with crushed ice	Both full legs (from gluteal to the foot)	*n* = 9 F—9 Age: 22 ± 3	Balance tests quiet standing, barefoot, 2 visual conditions, eyes open and closed, Two positions: Romberg position (large support base) Modified Tandem position (narrow support base) Duration: 30 s took to analysis, 3 times 2 min rest between trials V-root mean square (RMS), mean velocity (MV), sway area (SA), mean power frequently (MPF) of COP muscle temp. (1 cm below subcutaneous fat layer-vastus lateralis), skin temp. (2 sites of vastus lateralis and soleus), and core temp. were measured	Force plate^g^	*P* > 0.05 for all variables

Wassinger et al., 2007 [17]	20 min cubed ice ice bag 1.15 l, 1500 g The middle of the ice was centered over the acromion Data collection: before 2 times, after once	Shoulder dominant extremity	*n* = 22 M—14 F—8 Age: 21.6 ± 2.4	A-JPS P: standing (1) 20 FLEX-20° of shoulder flexion with 0° of humeral rotation and full elbow extension (2) 90 ABDER-90° of shoulder abduction with 90° of external rotation and full elbow extension Test: angle reproduction from 1 to 2 and from 2 to 1 position Blindfolded 3 trials in each direction V-aiming error [cm] functional ability of shoulder test V-functional throwing performance test-FTPI	Biodex System 3^h^	*P* > 0.05 all variables for JPS FTPI **P** < 0.001

Hart et al., 2005 [54]	20 min cubed ice (2 cm^3^/cub) ~0.5–1 kg Plastic bag: 24.5 × 45.7 cm anterior surface of the knee joint Data collection: before, after, 15 min and 30 min after	Knee chosen by subject	*n* = 20 M—9 F—11 Age: 23.8 ± 3.6	5 baseline forward jumps, Single leg landing at least 30 s rest between trials avg. from 5 trials V-F ground reaction forces [N/kg], sagittal knee joint motion [°], RMS of EMG [V]: gluteus medius, biceps femoris, vastus lateralis, medial gastrocnemius	Electric goniometer ELGOM^i^ Force plate^j^ EMG	*P* > 0.05 for all variables

Dover and Powers, 2004 [15]	30 min cubed ice, 1 kg Ice bag: 20 × 25 cm over the tip of the acromion Data collection: before, after	Shoulder dominant extremity	*n* = 30 M—15 F—15 Age: 22.2 ± 3.4	A-JPS P: standing with 90° shoulder abduction and 90° elbow flexion, the target angle position: 90% of internal (IR) and external rotation (ER), time to memorize 3 s 3 trials in each direction Blindfolded V-absolute error, variable error, constant error [°]	Inclinometer	*P* = 0.181 for all variables

Rubley et al., 2003 [24]	15 min ice-bath immersion Temp. of water: 10°C ~2,54 cm arm proximal to the medial epicondyle to the distal end of finders Data collection: before, after, and 15 min after	2x both arms	*n* = 15 M—8 F—7 Age: 22 ± 3	Force sense test P: sitting comfortably in a chair in front of a table (1) MIVC (10 s, 3 trials), pinch force measurements between thumb and index finger (2) Sensation tests: cutaneous sensation [g] 2-point discrimination testing [mm] (3) 10, 25, 40% of MIVC 5 trials, 30 s, 30 s break V-RMS of force; avg. and sd [N]	Force grip bars^k^ Monofilaments system^l^ Disk-Criminator^m^	Sensation tests **P** = 0.01 2-point discrim. *P* > 0.05 *P* > 0.05 for forces (precision and accuracy)

Uchio et al., 2003 [53]	15 min Icing System 2000^n^ maintains temp. 4°C of cooling pad Data collection: before, after, and 15 min after	Knee 2 days later other knee	*n* = 20 M—10 F—10 Age: 24.5	A-JPS P: seated 90° of knee flexion, leg was stopped at 10 different target angles between 5 and 25° knee flexion time to memorize 3 s, 10°/s velocity Blindfolded, 10 trials V-flexion error, extension error [°] Knee laxity test	Cybex^o^ dynamometer Knee arthrometer ^p^	**P** = 0.03

Tremblay et al., 2001 [29]	20 min crushed ice in a moist towel over the anterior aspects of the thigh Data collection: before, after	Quadriceps muscle dominant extremity	*n* = 20 M—14 F—6 Age: 22.1 ± 2.6	Weight-discrimination task P: seated of 90° flexion of the knee, movement: extension-flexion standard weight 2.5 kg Comparison weight: 0.5–0.4–0.28–0.11 kg Which weigh was heavier? 14 trials, blindfolded V-% of correct discrimination	Exercise table^q^ for lower extremity	*P* = 0.81

Hopper et al., 1997 [52]	15 min, ice water immersion, temp.: 4°C, to depth of 5 cm above the medial malleolus, data collection: before, after	Ankle (randomly chosen)	*n* = 49 M—42 F—7 Age: 19.4	A-JPS after passive placement at 40% and 80% of active full range of motion 3 trials in each section	Pedal goniometer	**P** = 0.049

Thieme et al., 1996 [51]	20 min 2 ice packs (30,5 × 49 cm), 1160 g of ice each One covered approx. 10 cm above to 10 cm below the patella, the other on and around popliteal space	Knee left leg	*n* = 37 M—21 F—16 Age: 23.4 ± 6.3	A-JPS P: seated, randomly assigned starting angles: 90, 60, or 30° of knee flexion, reproduce the movement for both the angles and timing of motion 90–60°, 60–30°, 30-full extension 2 trials in each sector Blindfolded V-peak angle, final angle [°]movement reproduction, total time of repetition [s]	Kin-Com Isokinetic dynamometer^r^	*P* > 0.05

LaRiviere and Osternig, 1994 [49]	5 and 20 min. ice immersion Temp.: 4°C, 4 cm distal from the knee joint line	Ankle	*n* = 31	A-JPS active reproduction after a predetermined angle was actively located, 2 joint angles: 30 and 40 of ankle flexion, 8 trials	Electrogoniometer	*P* > 0.05

Ingersoll et al., 1992 [48]	20 min cold water 1°C	Foot and ankle right leg	*n* = 21	One-leg balance test P: stork stand [s] Topagnosis test [cm] 2-point discrimination [cm]	A marker Stopwatch	*P* = 0.89

*Investigation instruments:*

^a^Biodex Balance System, Biodex Medical System, Inc. Shirley, New York

^b^AMTI Accusway, AMTI Copr., Watertown, MA

^c^Eagle 3D (5 cameras), Motion Analysis Corporation, Santa Rosa, CA, USA

^d^Ariel Performance Analysis System, Ariel Dynamics, CA, USA

^e^Cybex 770 Norm, Lumex Inc., Ronkonkoma, NY, USA

^f^KAT 3000, Breg., Vista, CA, USA

^g^Kistler 9261A, Winterthur, Switzerland

^h^Biodex System 3, Biodex Medical Inc., Shirley, NY, USA

^i^ELGOM, model SG110, Biometrics, Ltd., Ladysmith, VA

^j^Typ, 4060-10, Bertec, Inc., Columbus, Ohio

^k^Vishay Measurements Group, Shelton, CT

^l^Semmes-Weinstein monofilament, Lafayette Instruments, Lafayette, IN

^m^Lafayette Instruments, Lafayette, IN

^n^Icing System 2000, Nippon Sigmax, Japan

^o^Cybex International Inc., 10 Trotter Dr, Medway, MA 02053

^p^KT2000^TM^MEDmetric Corp, 7542 Trade St, San Diego, CA 92121-2412

^q^Model 2400, Midland Co, Columbia, SC

^r^Kin-Com, Isokinetic Dynamometer, Chattecx Corp., Hixson, TN

*Abbreviations:*

A-JPS: Active joint position sense

P-JPS: Passive joint position sense002E

PMR: Path of motion replication

LOS: Limits of stability

OKC: Open kinetic chain

CKC: Closed kinetic chain

*n*: Number of subjects

F: Female

M: Male

V: Dependent variable

P: Initial position

A/P: Anterior-posterior

M/L: Mediolateral

Age (mean, ±standard deviation).
